# Extracorporeal pediatric renal replacement therapy: diversifying application beyond kidney failure

**DOI:** 10.1007/s00467-024-06533-z

**Published:** 2024-10-08

**Authors:** Rahul Chanchlani, David Askenazi, Benan Bayrakci, Akash Deep, Jolyn Morgan, Tara M. Neumayr

**Affiliations:** 1 Department of Pediatrics, Division of Pediatric Nephrology, McMaster University, McMaster Children’s Hospital, Hamilton, ON Canada; 2 Department of Pediatrics, Division of Pediatric Nephrology, Pediatric and Infant Center for Acute Nephrology, Children’s of Alabama, University of Alabama at Birmingham, Birmingham, AL USA; 3 Department of Pediatric Intensive Care Medicine, The Center for Life Support Practice and Research, Hacettepe University, Ankara, Turkey; 4 Pediatric Intensive Care Unit, King’s College Hospital NHS Foundation Trust, London, UK; 5 Department of Women and Children’s Health, School of Life Course Sciences, King’s College London, London, SE1 7EH UK; 6 Center for Acute Care Nephrology, Cincinnati Children’s Hospital Medical Center, Cincinnati, OH USA; 7 Department of Pediatrics, Divisions of Pediatric Critical Care Medicine and Pediatric Nephrology, Washington University School of Medicine, St. Louis, MO USA

**Keywords:** Extra-renal, Continuous renal replacement therapy, Inborn errors of metabolism, Toxins, Liver failure, Tumor lysis syndrome, Trauma, Septic shock

## Abstract

The utilization of extracorporeal renal replacement therapy (RRT), including continuous renal replacement therapy (CRRT) and hemodialysis (HD), beyond the treatment of volume overload and acute kidney injury (AKI) has witnessed a significant shift, demonstrating the potential to improve patient outcomes for a range of diseases. This comprehensive review explores the non-kidney applications for RRT platforms in critically ill children, focusing on diverse clinical scenarios such as sepsis, inborn errors of metabolism, liver failure, drug overdose, tumor lysis syndrome, and rhabdomyolysis. In the context of sepsis and septic shock, RRT not only facilitates fluid, electrolyte, and acid/base homeostasis, but may offer benefits in cytokine regulation, endotoxin clearance, and immunomodulation which may improve multi-organ dysfunction as well as hemodynamic challenges posed by this life-threatening condition. RRT modalities also have an important role in caring for children with inborn errors of metabolism, liver failure, and tumor lysis syndrome as they can control metabolic derangements with the efficient clearance of endogenous toxins in affected children. In cases of drug overdose, RRT is a crucial tool for rapid extracorporeal clearance of exogenous toxins, mitigating potential organ damage. The intricate interplay between liver failure and kidney function is examined, elucidating the role of RRT and plasma exchange in maintaining fluid and electrolyte balance when hepatic dysfunction complicates the clinical picture. Furthermore, RRT and HD are explored in the context of rhabdomyolysis, highlighting their utility in addressing AKI secondary to traumatic events and crush syndrome.

**Graphical abstract:**

**Figure:**
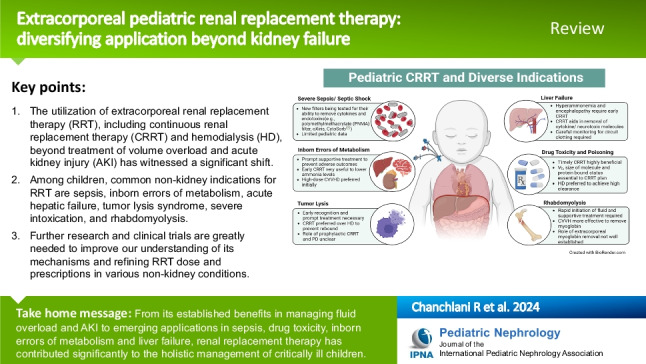
A higher resolution version of the Graphical abstract is available as Supplementary information

**Supplementary Information:**

The online version contains supplementary material available at 10.1007/s00467-024-06533-z.

## Introduction

**Fig. 1:**
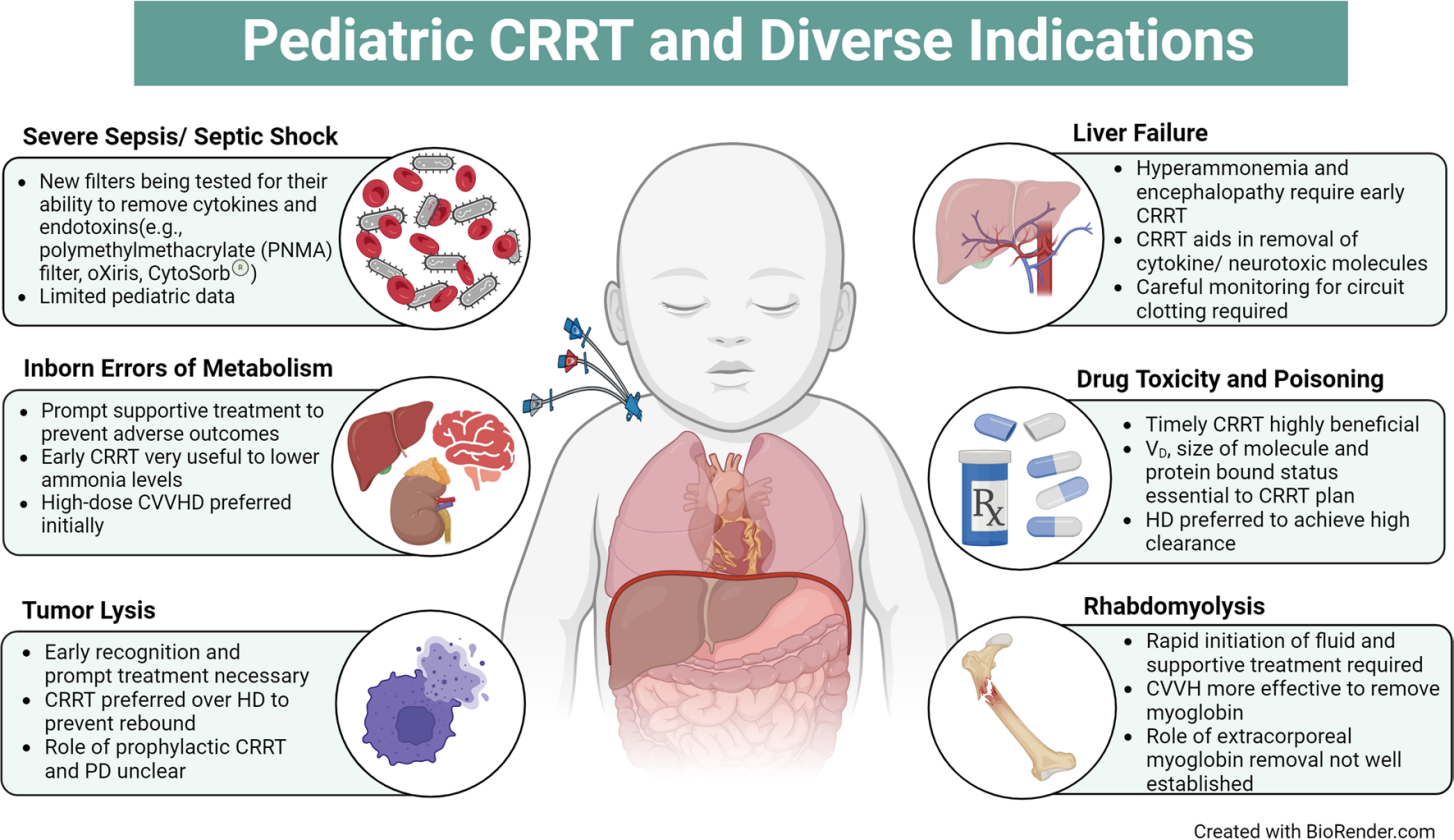
Diverse indications of renal replacement therapy in children

Extracorporeal renal replacement therapy, including CRRT and HD, are key components of managing acute kidney injury (AKI) and fluid overload among critically ill children. However, with the advancement of technology, there is an increase in the use of RRT and HD for non-kidney indications. Among children, common non-kidney indications for RRT are sepsis, inborn errors of metabolism, acute hepatic failure, tumor lysis syndrome, severe intoxication, and rhabdomyolysis (Fig. 1). In the US Prospective Pediatric Continuous Renal Replacement Therapy (ppCRRT) registry, between 2001 and 2005, 50 CRRT procedures were performed for non-kidney indications among 344 patients. The non-kidney indications included inborn errors of metabolism (42%), drug intoxication (36%), and tumor lysis syndrome (22%) [1]. Some patients with these syndromes (i.e., liver failure and sepsis) may require dialysis to help achieve the traditional goals of RRT (fluid and electrolyte homeostasis). Advanced approaches to care using novel filters and configurations may enhance clearance of inflammatory mediators and/or immunomodulation in patients with septic shock and elimination of exogenous or endogenous toxic solutes (such as inborn errors of metabolism, trauma, tumor lysis, or intoxication). 

This review provides a comprehensive overview of the application of CRRT and HD for various non-kidney indications in critically ill children, along with important technical nuances that should be considered when managing these patients.

## RRT in severe sepsis and septic shock

The proliferation of inflammatory mediators, cytokines, and endotoxins that characterize sepsis makes for a target-rich environment for the use of designed RRT filters. Treatment goals may include limiting the progression of multi-organ dysfunction syndrome (MODS), reversing systemic inflammatory response syndrome (SIRS), shortening the duration of organ dysfunction and reliance on invasive mechanical ventilation or inotropic support, reduction in ICU and hospital length of stay (LOS), and, of course, improvements in survival and lifelong functioning. The search for a filter that can achieve the removal of cytokines, endotoxin, or other harmful mediators was spurred by the dual observations that the AN-69 filter appeared to offer improved cytokine clearance in sepsis in comparison to other synthetic high-flux filters due to its particular adsorptive capacity and that, unfortunately, the AN-69 filter is associated with a higher risk of hemodynamic instability and collapse (the bradykinin-release syndrome) within the acidotic biochemical milieu common to septic patients [2, 3]. At present, several filters are being tested for their ability to impact outcomes in pediatric patients with severe sepsis, septic shock, and multi-organ dysfunction syndrome, which we will briefly summarize here. However, the effectiveness of RRT with or without these new filters in improving outcomes in children with sepsis is still under investigation.

Surface-treated membranes are now available for both targeted and traditional RRT applications. Surface treatment with polyethylenimine (PEI) aims to reduce the frequency and severity of membrane reactions and bradykinin-release syndrome by neutralizing the negative charge on the AN-69 membrane surface, which may improve the risk/benefit profile of these membranes.

The polymethylmethacrylate (PMMA) filter, with its notable adsorption of middle- and high-molecular-weight substances, was first developed in the 1970s but more recently has been leveraged for use in the treatment of endotoxin-mediated sepsis. A septic animal model was developed and demonstrated modulation of immunologic dysfunction via reduced tissue and systemic complement activation, thereby limiting kidney damage and fibrosis [4]. The PMMA filter has also been invoked in the treatment of COVID-19 and other inflammatory conditions such as acute pancreatitis [5, 6]. Robust trials of its use in clinical settings, including in pediatric patients, however, have not been published, and will be necessary before use of the PMMA filter can be widely advocated.

The oXiris membrane® (Gambro) is coated with heparin and PEI, a multilayer cationic complex that can adsorb negatively charged endotoxins on the surface while also trapping inflammatory cytokines in the bottom layer. A recent meta-analysis of 14 adult studies of the oXiris filter demonstrated reductions in 28-day mortality and ICU LOS, associated with lower Sequential Organ Failure Assessment (SOFA) scores, norepinephrine doses, interleukin-6 (IL-6) and lactate levels, and 7- and 14-day mortality, 90-day mortality, ICU and hospital mortality, and hospital LOS. However, they were not significantly different in the oXiris group compared to controls. Moreover, the evidence was deemed to be of low or very low level of certainty, as the analysis was only able to include 4 randomized controlled trials (RCTs), all of which had small sample sizes—152 subjects in the 4 studies combined, with the largest studies enrolling 60 subjects each—and unclear risk of bias; the remaining 10 studies were observational [7]. Published pediatric experience with the oXiris® filter is limited to one case series and several case reports, two of these describing the use of the filter in children with critical illness due to COVID-19 [8–11]. While the case reports suggest the possibility of dramatic improvements with the use of the oXiris® filter, the case series demonstrates a more variable response, with eight patients failing to improve during the course of treatment, five of whom ultimately died, and a high rate of mortality even among the patients who did show clinical improvement during oXiris® treatment [10].

The CytoSorb® filter has been employed for cytokine adsorption in a variety of inflammatory states due to its capacity to remove hydrophobic molecules under 55 kDa from the circulation. A recent systematic review and meta-analysis was unable to demonstrate a positive benefit from the use of the filter in a diagnostically heterogenous pooled patient population, and there was perhaps a negative effect on survival in the post-cardiac arrest subgroup [12]. Moreover, the observational studies in the meta-analysis all showed a moderate to serious risk of bias. In the subgroup of patients with sepsis, no difference in mortality was observed. Likewise, in the sub-analysis of the two RCTs in septic patients, the difference in mortality was not significant between the CytoSorb® and non-CytoSorb® groups, with a trend toward harm in the CytoSorb® group. No difference was demonstrated either broadly or within the sepsis subgroup in secondary outcomes such as LOS, norepinephrine use, or mean arterial pressure [12]. A pilot study of the CytoSorb® filter in pediatric patients with septic shock was published in 2023 (the PedCyto study), demonstrating improvements in the vasoactive medication usage and organ dysfunction scores and a trend toward lower 28-day mortality [13]. We eagerly await the results of the larger PedCyto study (NCT05658588) as we continue to evaluate the risks and benefits of CytoSorb® use in our patient population.

The Selective Cytopheretic Device (SCD) has been studied in 16 critically ill children > 15 kg with multi-organ dysfunction, including AKI requiring RRT. The device is designed to immunomodulate activated circulating leukocytes via a sequestering membrane in concert with citrate, resetting dysregulated leukocyte activation kinetics: monocytes become trapped and, under hypocalcemic conditions, change their phenotypes, while neutrophils undergo apoptosis. Its safety and feasibility in children were demonstrated in a study published in 2021 [14]. In 2024, the FDA granted a humanitarian device exemption approval for children > 10 kg with AKI due to sepsis or a septic condition requiring RRT. A prior adult RCT of similar design to the ongoing pediatric study demonstrated no significant difference in outcomes in the intention to treat analysis, but did show improved survival and reduced dialysis dependency in the subgroup of patients in whom the protocol’s recommended ionized calcium range (≤ 0.4 mmol/l) was maintained for at least 90% of the therapy time [15]. A randomized clinical trial (Neutralize AKI) in adults is currently underway.

As of April 2024, the Seraph100 Microbind Affinity Blood Filter (ExThera, Martinez, CA, USA) is authorized for emergency use to treat critically ill adult patients (≥ 18 years of age) with confirmed COVID-19 and established or imminent respiratory failure. The Seraph 100 Microbind filter is compatible and can be used with HD, CRRT, or in line with ECMO circuits. It acts as a surrogate to the patient’s heparin sulfan proteoglycans that line the endothelial glycocalyx that serves to bind pathogens. The filter uses heparin sulfate-coated microbeads within the filter to remove bacteria, viruses, fungi, and pathogenic molecules from the blood. Early reports of Seraph in adults show few side effects, and observational data suggest potential benefits to decrease bacteremia and shock and increase survival [16–18]. Two pediatric case reports, one involving an adolescent with COVID-19 and one involving an adolescent with a kidney transplant with adenovirus infection, have been reported [19, 20], as of February 2024.

## RRT in inborn errors of metabolism (IEM)

Urea cycle disorders, organic acidemias, fatty acid oxidation disorders, and mitochondrial cytopathies are metabolic emergencies, and prolonged elevation of ammonia or branched-chain amino acids (e.g., leucine) as well as severe lactic acidosis can cause profound hemodynamic consequences and irreversible neurological damage [21–23]. Prompt recognition and management of these conditions are crucial. A high index of suspicion for metabolic crisis is critical in children of any age with suggestive clinical symptoms [24]. Mortality rates remain as high as 25–50%, and non-survivors are likely sicker on admission [21, 25, 26].

Even before a definitive diagnosis is confirmed, emergency procedures for blood purification should commence. These include aggressive hydration as many of these babies have been sick and not feeding adequately for days. Stopping any form of protein and provision of high-dose dextrose concentration are essential to stop endogenous protein breakdown. Only limited evidence is available regarding the indication, best methods, and prescription for RRT. The precise plasma ammonia level that necessitates RRT remains a topic of debate and can be misleading [26, 27], but most experts agree that levels above 200 µmol/l cause direct brain damage [24, 28, 29]. Ultimately, the ongoing clinical condition is the main factor in deciding whether to initiate RRT [24].

Several factors influence the selection of the RRT method, including patient size, severity of enzyme deficiency, hemodynamic stability, local expertise, vascular access, and availability of extracorporeal devices. The decision for an appropriate RRT should be made jointly by nephrology, metabolic, and critical care teams.

Emergency initiation is required as the degree and duration of a hyperammonemic coma before initiating RRT are the primary prognostic factors [30]. While hemodialysis provides superior clearance rates, it comes with a notably increased risk of hypotension linked with HD [31, 32]. Even though CRRT offers reduced clearance rates, in small patients and large machines/filters, the clearance rates can approximate HD rates. The patient’s outcome may not be determined solely by the speed of ammonia clearance [33]. The continuous nature of CRRT avoids rebound hyperammonemia together with less dramatic fluid and osmotic shifts [25]. As preparations for RRT are underway, it is rational to consider ammonia-scavenging agents (sodium benzoate, sodium phenylacetate, sodium phenylbutyrate, glycerol phenylbutyrate), although they will be dialyzed along with other small molecules [26, 34, 35].

Recent guidelines for the management of hyperammonemia were published by a workgroup comprising an international expert panel of pediatric nephrologists. However, the consensus statements were formulated primarily based on case reports and retrospective studies and had low grade of evidence [24]. According to the guidelines, RRT is recommended for patients displaying rapidly deteriorating neurological conditions or cerebral edema, especially when blood ammonia levels surpass 150 µmol/l (256 µg/dl) or in the occurrence of moderate to severe encephalopathy [24]. Specifically, high-dose CVVHD (8000 ml/min/1.73 m^2^) is the recommended first-line treatment for severe hyperammonemia (blood ammonia level > 1000 µmol/l (1703 µg/dl)). Once ammonia concentrations drop below 200 µmol/l (280 µg/dl), transitioning to step-down CRRT is recommended [24]. During high-dose CRRT, careful attention should be paid to hypophosphatemia, hypokalemia, and hypocalcemia (if using citrate anticoagulation). In addition, replacing certain medications (specifically antiepileptics such as phenobarbitone, etc.) as well as nutrients and antibiotics which are cleared through dialysis is essential. While peritoneal dialysis offers a relatively slower ammonia removal, it becomes essential in contexts where extracorporeal treatments are not accessible or are deemed unsafe, ensuring that detoxification is not postponed.

During the acute crises of IEM, the role of RRT extends beyond just clearing toxic accumulations. It also aids in restoring pH and ensuring adequate caloric need without limiting fluid intake. The most common kidney phenotype in mitochondrial disease is proximal tubulopathy; however, all regions of the nephron can be affected. Additionally, mitochondrial fatty acid b-oxidation (FAO) defects frequently cause recurrent rhabdomyolysis leading to AKI. Considering the hazardous effect of IEMs on the kidney, RRT is also necessary for managing altered kidney function. A grave factor complicating the disease process is the increased risk of sepsis and secondary hemophagocytic syndrome. Thus, RRT might also serve an immunomodulatory function, addressing these hyper-inflammatory conditions during the acute crises [21].

## RRT in tumor lysis syndrome

In tumor lysis syndrome (TLS), the rapid breakdown of tumor cells occurs leading to the metabolic imbalance caused by the severe and abrupt release of cellular contents into the blood. TLS is an oncological emergency and occurs frequently in children and adults with various malignancies such as acute lymphoblastic leukemia and Burkitt’s lymphoma typically within 48–72 h after the initiation of chemotherapy, although it may also occur spontaneously in highly proliferative tumors or among patients with high tumor burden or sensitivity to chemotherapy. Rapid breakdown of cells leads to the release of intracellular contents at a high rate, thereby disrupting the homeostatic mechanisms and leading to hyperkalemia, hypocalcemia, hyperuricemia, uremia, and metabolic acidosis [36].

Cairo-Bishop criteria is commonly used to diagnose TLS [37] which suggests that *laboratory* TLS is present if > 2 of the following are present within 3 days before or up to 7 days following chemotherapy: elevated uric acid (> 8 mg/dl), elevated phosphate (> 6.5 mg/dl), elevated potassium (> 6 meq/l), and low calcium (< 7 mg/dl). *Clinical* TLS includes any one of the features like AKI (serum creatinine > 1.5 times the normal or oliguria for 6 h), or the presence of seizures, intracranial bleed, or cardiac arrhythmias. If not recognized in a timely fashion, TLS is life-threatening, and in-hospital mortality ranges between 15 and 30% among children and adults [38, 39]. AKI, especially AKI requiring dialysis, is an important predictor of adverse outcomes associated with TLS [40, 41]. Diagnosis requires a high level of suspicion, and prognosis may improve with anticipation and early recognition of metabolic and kidney complications in association with rapid institution of prophylactic and treatment measures.

Management and outcome data among children is limited [42]. Initial management includes close monitoring; vigorous hydration (1.5 to 2 times maintenance rate) to achieve a urine output of around 4 ml/kg/h for infants and 100 ml/m^2^/h for older children; use of rasburicase for hyperuricemia to prevent AKI from uric acid deposition in tubules; use of diuretics; and management of hyperkalemia, hyperphosphatemia, and hypocalcemia [38, 43]. Further details on these management strategies have been published recently [39, 43]. However, some patients may require RRT due to severe AKI or electrolyte imbalance, and oliguria.

The choice of dialysis depends on the hemodynamic condition of the patient, fluid status, and presence of multi-organ involvement as well as the rate of malignant cell turnover. Due to the expected ongoing lysis of tumor cells and the risk of rebound hyperkalemia and hyperphosphatemia, CRRT is preferred [38]. A recent study demonstrated that CRRT is safe among children with hematological malignancies with severe TLS and reverses electrolyte and metabolic abnormalities within 6–12 h [44]. However, among children with severe hyperkalemia and hyperuricemia, intermittent HD (or high-dose CRRT) is recommended initially to be switched to standard-dose CRRT. Dialysis should continue until there is sufficient recovery of kidney function and/or urine output and resolution of severe electrolyte abnormalities [38]. The role of prophylactic RRT is unclear, and larger studies are needed to assess its benefit [45]. Peritoneal dialysis is not recommended for TLS.

## RRT for liver failure

Another important non-kidney indication of RRT is in children with liver failure. Liver failure can be acute, chronic, or acute on chronic liver failure. The pathophysiologic derangements are different in each of these entities; therefore, the indications, rationale, and logistics will be different as well. For the purposes of this section, we will be discussing the non-kidney use of RRT in patients with pediatric acute liver failure (PALF).

Extracorporeal therapies form an important part of the multi-modal treatment armamentarium in children with ALF. An ideal extracorporeal device should be able to perform both the synthetic and detoxification functions of the liver. Though not strictly a liver assist device, RRT is the most used modality in clinical practice due to the familiarity with the device in pediatric ICUs across the globe. Since one of the main causes of mortality in these children is raised intracranial pressure secondary to impaired detoxification of toxic molecules, especially ammonia, RRT can be used either as a bridge to spontaneous liver recovery or successful liver transplantation although data on its impact on survival in these patients are sparse.

Early use of RRT is recommended. Hepatic encephalopathy (HE) grades 3 and 4 and hyperammonemia (150 µmol/l (256 µg/dl) are two of the non-kidney indications for the commencement of RRT. However, the patient’s clinical condition and prognosis should also be considered. We recommend starting RRT early for hyperammonemia, fluid control, and nutrition. In a single-center study from Europe, it was found that, on average, the likelihood of survival increased by 50% for every 10% decrease in ammonia from baseline at 48 h. There was an earlier initiation of RRT (time from PICU admission) in survivors. Change in practice to early initiation of high-volume CRRT led to increased survival. After excluding children who underwent liver transplantation, CRRT led to better survival [46].

The primary aim of RRT in PALF is to decrease the levels of circulating cytokines and other neurotoxic molecules rapidly to decrease systemic inflammation and cerebral edema. High-volume CRRT removes cytokines such as TNF-α and IL-1β, which are also implicated in the pathogenesis of ALF and HE. A report from Paris on high-volume hemofiltration (HVHF) in 22 children with PALF showed significant improvements in hemodynamics at 24 h and a decreased grade of HE at 48 h [47]. However, one must be mindful of the removal of drugs and nutrients while increasing the dose of CRRT.

Studies to determine superiority among CVVH vs. CVVHD or CVVHDF in PALF have not been conducted. It would depend upon institutional practice and staff familiarity, although, ammonia being a small molecule and cytokines being middle molecules, CVVHDF theoretically offers optimal conditions for prolonged filter life with dialysis, while adding middle molecule convective clearance. In PALF, CRRT causes an equimolar decrease of both pro- and anticoagulant factors, leading to a rebalanced hemostasis. Endogenous thrombin generation potential in these patients has been shown to be higher than those without ALF [48]. Hence, these patients can be functionally prothrombotic, and there is a higher chance of circuit/filter clotting when using CRRT. Decreasing downtimes in these patients is of paramount importance as the longer the downtime, the less effective CRRT will be at removing ammonia and other neurotoxins, and hence, there can be a greater risk of cerebral edema. Therefore, it is vital to keep these circuits going by using safe and effective anticoagulants. The choices are unfractionated heparin, regional citrate anticoagulation with close monitoring for citrate lock and citrate accumulation, platelet inhibitors like prostacyclin, and nafamostat mesylate. In the recently published data, use of prostacyclin was shown to be safe (major/minor bleeding, hypotension), efficacious (effective filter life, solute clearance, and effective 60-h filter life), and cost effective [49, 50].

Another modality that requires special mention is total plasma exchange (TPE) in children with ALF. TPE is cited as Level 1 evidence by the European Association for Study in Liver disease (EASL) as well as the American Society for Apheresis for use in ALF [51, 52]. High-volume plasma has been shown to improve transplant-free survival by immune modulation leading to amelioration of multi-organ failure [53]. TPE and CRRT can be used in tandem during the treatment of PALF with the advantage that CRRT does not have to be stopped, thereby decreasing downtime and minimizing circuit changes. Increasingly, CRRT is being used intra-operatively during liver transplant especially in those children who are on CRRT pre-operatively for severe AKI, those with evidence of raised intracranial pressure, and those in multi-organ failure.

Finally, SPAD and MARS have been used to eliminate protein-bound toxins in PALF though effects on survival have not been proven [54].

## RRT in drug toxicity and poisoning

Another non-kidney indication which can become life-threatening is the ingestion of drugs or environmental substance for which timely RRT can be highly beneficial. Extracorporeal therapies can remove some toxins rapidly, and thus may be urgently utilized to reduce the morbidity and mortality from toxicities. The properties that make a substance amenable to removal and approaches to choosing different extracorporeal modalities are briefly discussed below. A review of these topics has been published recently [55].

When considering extracorporeal therapy for the clearance of intoxications, an understanding of the three inherent properties of the substance is essential. First, a substance can be removed with hemodialysis/hemofiltration only if it is localized to the intravascular space. A drug’s volume of distribution (*V*_D_) is the volume in which the substance is distributed within the body’s intravascular and extravascular spaces. The lower the *V*_D_, the higher its availability for extracorporeal removal [56]. Second, only the portion of the substance which is *not* bound to protein can be cleared with dialysis. If a substance has very high protein binding, it is hard to dialyze the large molecular weight drug–protein complex [57]. Importantly, when evaluating the percent protein binding of a substance during intoxication, protein binding is saturable such that at toxic levels the percentage of unbound drug available is higher because of the saturation of protein binding sites [58]. Finally, the size of the pores of the filter has an important role in substance removal as only those substances that are smaller than the pore sizes can pass through the filter membrane [59]. Typical membranes used in hemodialysis can remove substances that are up to 15 kDa. Because convective clearances drag solvents across the membrane, hemofiltration mode on a CRRT circuit can remove “middle” molecules (close to 50 kDa) [60–62]. Novel membranes that have higher membrane cut-offs may enhance dialysis clearance of certain substances.

Hemodialysis is by far the most used and available modality for ingestions. For a substance that has a low *V*_D_, has low protein binding, and is small enough to be dialyzed through the membrane, hemodialysis is the most efficient approach. High-dose CRRT in small children using adult-sized filters and machines can allow clearances that approximate HD.

When prescribing HD for intoxications, a high blood flow rate and dialysis flow rate should be maximized to optimize clearance. Dialysis has historically been used for lithium, salicylates, and alcohols that are toxic. In addition, case reports of dialysis for carbamazepine, valproic acid, and vancomycin have been reported [60, 63, 64]. The main drawback to hemodialysis is that once the therapy stops, the drug concentrations may rebound as the substrate within the interstitial and cellular space will then redistribute into the intravascular space. To address this rebound, frequent intermittent treatment can be employed. Others choose a two-step approach whereby an intermittent HD treatment is performed to bring the level down rapidly, and then, CRRT is employed to keep the level from rebounding. In small children, high-dose CRRT (to bring the levels down quickly) and, then once at the target level, a lower dose that maintains the level can be used to avoid rebound. Other potential advantages to CRRT are the ability to use a convection-based approach for higher middle molecule clearance, tighter fluid control, and tighter sodium control (which is important in patients at risk of rapid sodium drop or cerebral edema). Using CKRT yields a lower hourly efficiency; however, the overall daily clearance is higher with continuous drug elimination.

Therapeutic plasma exchange (TPE) has the unique advantage of being able to remove drugs and/or drug-protein complexes of high molecular size. TPE that uses centrifuge techniques can remove any drug in the plasma (whether it is protein-bound or not). The pore size of TPE that uses filtration techniques is much higher than standard dialysis membranes which also allows very high clearance of molecules that are highly protein-bound and/or large in size.

Single-pass albumin dialysis (SPAD) can enhance the clearance of protein-bound drugs in the context of a CRRT system. SPAD employs the addition of albumin to the dialysis fluid which can help enhance clearance of certain protein-bound drugs by shifting the gradient of the free portion of the drug being cleared. Case reports of drug clearance with SPAD have been published for carbamazepine, methotrexate, acetaminophen, diltiazem, and phenobarbital [65–70] with albumin concentration into the dialysis fluid of 2–4 g/dl. While mechanistically appropriate, this approach may be costly, and supply prohibited given the high albumin volume that is needed.

The molecular absorbent recirculating system (MARS®) encompasses a CRRT circuit in tandem with an albumin circuit that recirculates the albumin through a charcoal filter. This charcoal filter is an anion exchange column. FDA approved in 2013 for certain intoxications, successful use of MARS has been reported for ingestions of toxic mushrooms, diazepam, phenytoin, heavy metals, lamotrigine, and theophylline [71–75].

## RRT in patients with rhabdomyolysis

In rhabdomyolysis, rapid disruption of skeletal muscle occurs leading to a release of large amounts of myoglobin in the bloodstream. Rhabdomyolysis is most often caused by direct traumatic injury; however, it can also occur due to drugs, toxins, infections, electrolyte and metabolic disorders, genetic disorders, crush syndrome, neuroleptic malignant syndrome, and malignant hyperthermia. Here, rhabdomyolysis due to traumatic injury and crush syndrome is discussed.

Adults and children are at risk for developing rhabdomyolysis and AKI following a traumatic injury and crush syndrome [76–79]. The development of AKI may be due to severe blood loss resulting in inadequate kidney perfusion, rhabdomyolysis from tissue death, a direct kidney injury, abdominal compartment syndrome, or from the nephrotoxic effects of agents used to diagnose and treat injuries [79–82]. These events can trigger skeletal muscle necrosis wherein intracellular metabolites (e.g., urea, potassium, phosphorus) and proteins (e.g., creatinine kinase and myoglobin) are released into the bloodstream. This response coupled with profuse uncontrolled bleeding, coagulopathy, and/or infection may lead to life-threatening multisystem organ failure and severe kidney dysfunction [79, 80].

Rapid fluid replacement therapy is necessary to avoid hypovolemia. Fluid losses can be remarkable; hence, it is crucial to keep a positive fluid balance. In addition to intense hydration, management of traumatic injury and crush syndrome involves urine alkalization and sometimes kidney replacement therapy. It is essential to maintain a urine pH at ≥ 6.5 to avoid the formation of uric acid crystals and alkaline urine also increases dissolution of myoglobin and avoids further deposition.

Multiple studies suggest that early initiation of CRRT may have a favorable impact on survival outcomes for traumatized adult patients with AKI [80, 83, 84]. A single study revealed that 57% of adult patients developed AKI with 13% requiring CRRT [76]. Unfortunately, pediatric data describing trauma-induced AKI and the utility of CRRT are scarce. However, a recent systematic review and meta-analysis conducted by Yousefifard and colleagues found that AKI increased the risk of mortality by about fivefold for traumatized children when compared to those without AKI [79]. The prevalence of AKI was more likely to occur following mild to severe direct kidney trauma or rhabdomyolysis from earthquake-related injuries (12.64% and 24.60%, respectively).

In the setting of rhabdomyolysis, studies have shown that convective therapy is more effective at removing middle and large molecules such as myoglobin when compared to diffusive therapy [85]. In general, the timing of RRT remains up for debate; however, early initiation may be useful in eliminating toxic substances, managing fluid overload, and preventing further fluid accumulation, all of which can further exacerbate an already severely compromised state. It seems plausible to say that initiation of CRRT can aid in the prevention and/or mitigation of adverse outcomes. Future work with a specific focus related to the timing of CRRT for children in the setting of trauma/crush syndrome-induced AKI and the impact on outcomes is greatly needed. PD offers less clearance but has the advantage of not requiring a vascular access or water and electricity infrastructures in disaster conditions.

Considering the pathogenic role of high-dose myoglobin exposure to the kidneys, extracorporeal myoglobin removal is often considered in clinical practice. High cut-off membranes (i.e., Theralite®) are efficient in myoglobin removal but are associated with significant albumin loss. Accounting for myoglobin’s molecular size (18 kDa), medium cut-off membranes (i.e., Theranova®) minimize this risk. Cytokine adsorber membranes are also an attractive option for myoglobin removal but adsorber saturation could be a problem limiting its efficacy. The role of extracorporeal removal of myoglobin in the treatment of rhabdomyolysis is not yet fully established [86].

## Conclusions

The expanding role of RRT in non-kidney indications presents a promising frontier in critical care medicine. From its established benefits in managing fluid overload and AKI to emerging applications in sepsis, drug toxicity, inborn errors of metabolism, and liver failure, RRT has contributed significantly to the holistic management of critically ill children. Further research and clinical trials are greatly needed to improve our understanding of its mechanisms and refine RRT doses and prescriptions in various non-kidney conditions to optimize patient outcomes and enhance the quality of care in diverse clinical scenarios. Additionally, research to understand optimal medication dosing in the context of high dose and shifting RRT clearance rates is imperative.

## Supplementary Information

Below is the link to the electronic supplementary material.

Graphical abstract (PPTX 646 KB)

## References

[1] Fleming GM, Walters S, Goldstein SL, Alexander SR, Baum MA, Blowey DL, Bunchman TE, Chua AN, Fletcher SA, Flores FX, Fortenberry JD, Hackbarth R, McBryde K, Somers MJ, Symons JM, Brophy PD (2012) Nonrenal indications for continuous renal replacement therapy: a report from the Prospective Pediatric Continuous Renal Replacement Therapy Registry Group. Pediatr Crit Care Med 13:e299-304 22805158 10.1097/PCC.0b013e31824fbd76

[2] Thomas M, Moriyama K, Ledebo I (2011) AN69: evolution of the world’s first high permeability membrane. Contrib Nephrol 173:119–129 21865784 10.1159/000328961

[3] Monard C, Rimmelé T, Ronco C (2019) Extracorporeal blood purification therapies for sepsis. Blood Purif 47(Suppl 3):1–14 30974444 10.1159/000499520

[4] Stasi A, Franzin R, Divella C, Sallustio F, Curci C, Picerno A, Pontrelli P, Staffieri F, Lacitignola L, Crovace A, Cantaluppi V, Medica D, Ronco C, de Cal M, Lorenzin A, Zanella M, Pertosa GB, Stallone G, Gesualdo L, Castellano G (2021) PMMA-based continuous hemofiltration modulated complement activation and renal dysfunction in LPS-induced acute kidney injury. Front Immunol 12:605212 33868226 10.3389/fimmu.2021.605212 PMC8047323

[5] Spasiano A, De Luca G, Bartoli G, Dello Strologo A, Arena M, Grandaliano G (2024) COVID-19: the dysregulated response to infection - why consider polymethylmethacrylate membrane in hemodialysis patients? Blood Purif 53:373–378 37844557 10.1159/000533738 PMC11412687

[6] Kinjoh K, Nagamura R, Sakuda Y, Yamauchi S, Takushi H, Iraha T, Idomari K (2022) Clinical efficacy of blood purification using a polymethylmethacrylate hemofilter for the treatment of severe acute pancreatitis. Acute Crit Care 37:398–406 35791651 10.4266/acc.2022.00192 PMC9475162

[7] Wang G, He Y, Guo Q, Zhao Y, He J, Chen Y, Chen W, Zhou Y, Peng Z, Deng K, Guan J, Xie W, Chang P, Liu Z (2023) Continuous renal replacement therapy with the adsorptive oXiris filter may be associated with the lower 28-day mortality in sepsis: a systematic review and meta-analysis. Crit Care 27:275 37424026 10.1186/s13054-023-04555-x PMC10331993

[8] Phan PH, Nguyen DT, Dao NH, Nguyen HTT, Vu AV, Hoang ST, Nguyen LV, Cao TV, Tran DM (2022) Case report: successful treatment of a child with COVID-19 reinfection-induced fulminant myocarditis by cytokine-adsorbing oXiris® hemofilter continuous veno-venous hemofiltration and extracorporeal membrane oxygenation. Front Pediatr 10:946547 35903158 10.3389/fped.2022.946547 PMC9315247

[9] Lalwani P, Baskaran S, Uribe DA, Ramaiah A, Saqib A, ElMesserey M, Fathi EM, Tabata Y, Fink C, Pallavidino M (2022) A case of COVID-19-associated pediatric multisystem inflammatory syndrome in shock managed by cytokine filtration. Case Rep Pediatr 2022:3373289 35127192 10.1155/2022/3373289 PMC8808292

[10] Morin L, Charbel R, Cousin VL, Marais C, Claude C, Barreault S, Durand P, Miatello J, Tissières P (2023) Blood purification with oXiris© in critically ill children with vasoplegic shock. Blood Purif 52:541–548 37105135 10.1159/000530147

[11] Ying J, Cai X, Lu G, Chen W (2023) The use of membranes (ST-100, oXiris, and M60) for continuous renal replacement therapy in a child with sepsis. Case Rep Crit Care 2023:2000781 37324650 10.1155/2023/2000781 PMC10264131

[12] Becker S, Lang H, Vollmer Barbosa C, Tian Z, Melk A, Schmidt BMW (2023) Efficacy of CytoSorb®: a systematic review and meta-analysis. Crit Care 27:215 37259160 10.1186/s13054-023-04492-9 PMC10230475

[13] Bottari G, Guzzo I, Cappoli A, Labbadia R, Perdichizzi S, Serpe C, Creteur J, Cecchetti C, Taccone FS (2023) Impact of CytoSorb and CKRT on hemodynamics in pediatric patients with septic shock: the PedCyto study. Front Pediatr 11:1259384 37780052 10.3389/fped.2023.1259384 PMC10540853

[14] Goldstein SL, Askenazi DJ, Basu RK, Selewski DT, Paden ML, Krallman KA, Kirby CL, Mottes TA, Terrell T, Humes HD (2021) Use of the selective cytopheretic device in critically ill children. Kidney Int Rep 6:775–784 33732992 10.1016/j.ekir.2020.12.010 PMC7938071

[15] Tumlin JA, Galphin CM, Tolwani AJ, Chan MR, Vijayan A, Finkel K, Szamosfalvi B, Dev D, DaSilva JR, Astor BC, Yevzlin AS, Humes HD (2015) A multi-center, randomized, controlled, pivotal study to assess the safety and efficacy of a selective cytopheretic device in patients with acute kidney injury. PLoS One 10:e0132482 26244978 10.1371/journal.pone.0132482 PMC4526678

[16] Chitty SA, Mobbs S, Rifkin BS, Stogner SW, Lewis MS, Betancourt J, DellaVolpe J, Abouzahr F, Wilhelm AM, Szerlip HM, Parikh A, Gaeta RM, Rivera I, Park C, Levi B, Anesi GL, Alcover KC, Arnold TB, Howard JT, Sharma K, Pratt KP, Stewart IJ, Chung KK (2022) A multicenter evaluation of the Seraph 100 Microbind Affinity Blood Filter for the treatment of severe COVID-19. Crit Care Explor 4:e0662 35506015 10.1097/CCE.0000000000000662 PMC9049035

[17] Schmidt JJ, Borchina DN, Van’t Klooster M, Bulhan-Soki K, Okioma R, Herbst L, Rodríguez DS, Premužić V, Büttner S, Bader B, Serednicki W, Zasada E, Schmitz M, Quabach RA, Hrincheva M, Fühner T, Kielstein JT (2022) Interim analysis of the COSA (COVID-19 patients treated with the Seraph® 100 Microbind® Affinity filter) registry. Nephrol Dial Transplant 37:673–680 34875087 10.1093/ndt/gfab347 PMC8689741

[18] Stoffel S, Boster J, Jarrett Z, Rosas M, Kalra A, Nugyen M, Morris M, Walter R (2023) Single-center experience with the Seraph-100® Microbind® Affinity Blood Filter in patients with SARS-CoV-2 infection and septic shock at a military treatment facility. Mil Med 188:e2670–e2674 36852879 10.1093/milmed/usad063

[19] Merrill KA, Krallman KA, Loeb D, Standage SW, Mattoon D, Shan D, Goldstein SL, Schuh MP (2023) First-time use of the Seraph(®) 100 Microbind(®) Affinity Blood Filter in an adolescent patient with severe COVID-19 disease: a case report. Case Rep Nephrol Dial 13:1–6 36741548 10.1159/000527290 PMC9891843

[20] Li DS, Burke TM, Smith JM, Reed RC, Okamura DM, Menon S (2024) Use of the Seraph® 100 Microbind® Affinity Blood Filter in an adolescent patient with disseminated adenoviral disease. Pediatr Nephrol 39:331–335 37505308 10.1007/s00467-023-06097-4

[21] Yetimakman AF, Kesici S, Tanyildiz M, Bayrakci B (2019) Continuous renal replacement therapy for treatment of severe attacks of inborn errors of metabolism. J Pediatr Intensive Care 8:164–169 31402993 10.1055/s-0039-1683991 PMC6687454

[22] Aygun F, Aygun D, Erbek Alp F, Zubarioglu T, Zeybek C, Cam H (2018) The impact of continuous renal replacement therapy for metabolic disorders in infants. Pediatr Neonatol 59:85–90 28778517 10.1016/j.pedneo.2017.04.004

[23] Eminoglu FT, Oncul U, Kahveci F, Okulu E, Kraja E, Kose E, Kendirli T (2022) Characteristics of continuous venovenous hemodiafiltration in the acute treatment of inherited metabolic disorders. Pediatr Nephrol 37:1387–1397 34693482 10.1007/s00467-021-05329-9 PMC8542505

[24] Raina R, Bedoyan JK, Lichter-Konecki U, Jouvet P, Picca S, Mew NA, Machado MC, Chakraborty R, Vemuganti M, Grewal MK, Bunchman T, Sethi SK, Krishnappa V, McCulloch M, Alhasan K, Bagga A, Basu RK, Schaefer F, Filler G, Warady BA (2020) Consensus guidelines for management of hyperammonaemia in paediatric patients receiving continuous kidney replacement therapy. Nat Rev Nephrol 16:471–482 32269302 10.1038/s41581-020-0267-8 PMC7366888

[25] Ames EG, Powell C, Engen RM, Weaver DJ Jr, Mansuri A, Rheault MN, Sanderson K, Lichter-Konecki U, Daga A, Burrage LC, Ahmad A, Wenderfer SE, Luckritz KE (2022) Multisite retrospective review of outcomes in renal replacement therapy for neonates with inborn errors of metabolism. J Pediatr 246:116-122.e111 35358588 10.1016/j.jpeds.2022.03.043 PMC9233075

[26] Ames EG, Luckritz KE, Ahmad A (2020) A retrospective review of outcomes in the treatment of hyperammonemia with renal replacement therapy due to inborn errors of metabolism. Pediatr Nephrol 35:1761–1769 32232638 10.1007/s00467-020-04533-3

[27] Gupta S, Fenves AZ, Hootkins R (2016) The role of RRT in hyperammonemic patients. Clin J Am Soc Nephrol 11:1872–1878 27197910 10.2215/CJN.01320216 PMC5053785

[28] Picca S, Dionisi-Vici C, Abeni D, Pastore A, Rizzo C, Orzalesi M, Sabetta G, Rizzoni G, Bartuli A (2001) Extracorporeal dialysis in neonatal hyperammonemia: modalities and prognostic indicators. Pediatr Nephrol 16:862–867 11685590 10.1007/s004670100702

[29] Msall M, Batshaw ML, Suss R, Brusilow SW, Mellits ED (1984) Neurologic outcome in children with inborn errors of urea synthesis. Outcome of urea-cycle enzymopathies. N Engl J Med 310:1500–1505 6717540 10.1056/NEJM198406073102304

[30] Batshaw ML (1984) Hyperammonemia. Curr Probl Pediatr 14:1–69 6510017 10.1016/0045-9380(84)90047-1

[31] Kaneko M, Ogasawara K, Go H, Imamura T, Momoi N, Hosoya M (2013) Continuous hemodialysis therapy for an extremely low-birthweight infant with hyperammonemia. Pediatr Int 55:656–658 24134757 10.1111/ped.12101

[32] Davenport A, Will EJ, Davison AM (1990) Early changes in intracranial pressure during haemofiltration treatment in patients with grade 4 hepatic encephalopathy and acute oliguric renal failure. Nephrol Dial Transplant 5:192–198 2113646 10.1093/ndt/5.3.192

[33] Picca S, Dionisi-Vici C, Bartuli A, De Palo T, Papadia F, Montini G, Materassi M, Donati MA, Verrina E, Schiaffino MC, Pecoraro C, Iaccarino E, Vidal E, Burlina A, Emma F (2015) Short-term survival of hyperammonemic neonates treated with dialysis. Pediatr Nephrol 30:839–847 25185886 10.1007/s00467-014-2945-x

[34] Cho H (2019) Renal replacement therapy in neonates with an inborn error of metabolism. Korean J Pediatr 62:43–47 30404428 10.3345/kjp.2018.07143 PMC6382961

[35] Bunchman TE, Barletta GM, Winters JW, Gardner JJ, Crumb TL, McBryde KD (2007) Phenylacetate and benzoate clearance in a hyperammonemic infant on sequential hemodialysis and hemofiltration. Pediatr Nephrol 22:1062–1065 17277951 10.1007/s00467-007-0436-z

[36] Wilson FP, Berns JS (2014) Tumor lysis syndrome: new challenges and recent advances. Adv Chronic Kidney Dis 21:18–26 24359983 10.1053/j.ackd.2013.07.001 PMC4017246

[37] Cairo MS, Bishop M (2004) Tumour lysis syndrome: new therapeutic strategies and classification. Br J Haematol 127:3–11 15384972 10.1111/j.1365-2141.2004.05094.x

[38] Jones GL, Will A, Jackson GH, Webb NJ, Rule S (2015) Guidelines for the management of tumour lysis syndrome in adults and children with haematological malignancies on behalf of the British Committee for Standards in Haematology. Br J Haematol 169:661–671 25876990 10.1111/bjh.13403

[39] Stephanos K, Dubbs SB (2021) Pediatric hematologic and oncologic emergencies. Emerg Med Clin North Am 39:555–571 34215402 10.1016/j.emc.2021.04.007

[40] Darmon M, Guichard I, Vincent F, Schlemmer B, Azoulay E (2010) Prognostic significance of acute renal injury in acute tumor lysis syndrome. Leuk Lymphoma 51:221–227 20001238 10.3109/10428190903456959

[41] Garimella PS, Balakrishnan P, Ammakkanavar NR, Patel S, Patel A, Konstantinidis I, Annapureddy N, Nadkarni GN (2017) Impact of dialysis requirement on outcomes in tumor lysis syndrome. Nephrology (Carlton) 22:85–88 27119419 10.1111/nep.12806

[42] Flood K, Rozmus J, Skippen P, Matsell DG, Mammen C (2021) Fluid overload and acute kidney injury in children with tumor lysis syndrome. Pediatr Blood Cancer 68:e29255 34302706 10.1002/pbc.29255

[43] Cheung WL, Hon KL, Fung CM, Leung AK (2020) Tumor lysis syndrome in childhood malignancies. Drugs Context 9 10.7573/dic.2019-8-2 PMC7048108 32158483

[44] Anderson A, Shoulders L, James V, Ashcraft E, Cheng C, Ribeiro R, Elbahlawan L (2023) Benefit of continuous kidney replacement therapy for managing tumor lysis syndrome in children with hematologic malignancies. Front Oncol 13:1234677 37664024 10.3389/fonc.2023.1234677 PMC10471890

[45] Saccente SL, Kohaut EC, Berkow RL (1995) Prevention of tumor lysis syndrome using continuous veno-venous hemofiltration. Pediatr Nephrol 9:569–573 8580012 10.1007/BF00860936

[46] Deep A, Stewart CE, Dhawan A, Douiri A (2016) Effect of continuous renal replacement therapy on outcome in pediatric acute liver failure. Crit Care Med 44:1910–1919 27347761 10.1097/CCM.0000000000001826

[47] Chevret L, Durand P, Lambert J, Essouri S, Balu L, Devictor D, Tissieres P (2014) High-volume hemofiltration in children with acute liver failure. Pediatr Crit Care Med 15:e300-305 24901801 10.1097/PCC.0000000000000172

[48] Habib M, Roberts LN, Patel RK, Wendon J, Bernal W, Arya R (2014) Evidence of rebalanced coagulation in acute liver injury and acute liver failure as measured by thrombin generation. Liver Int 34:672–678 24164805 10.1111/liv.12369

[49] Deep A, Alexander EC, Khatri A, Kumari N, Sudheendhra K, Patel P, Joarder A, Elghuwael I (2024) Epoprostenol (prostacyclin analog) as a sole anticoagulant in continuous renal replacement therapy for critically ill children with liver disease: single-center retrospective study, 2010–2019. Pediatr Crit Care Med 25:15–23 38169336 10.1097/PCC.0000000000003371 PMC10756692

[50] Miyaji MJ, Ide K, Takashima K, Maeno M, Krallman KA, Lazear D, Goldstein SL (2022) Comparison of nafamostat mesilate to citrate anticoagulation in pediatric continuous kidney replacement therapy. Pediatr Nephrol 37:2733–2742 35348901 10.1007/s00467-022-05502-8

[51] Wendon J, Cordoba J, Dhawan A, Larsen FS, Manns M, Samuel D, Simpson KJ, Yaron I, Bernardi M (2017) EASL Clinical Practical Guidelines on the management of acute (fulminant) liver failure. J Hepatol 66:1047–1081 28417882 10.1016/j.jhep.2016.12.003

[52] Connelly-Smith L, Alquist CR, Aqui NA, Hofmann JC, Klingel R, Onwuemene OA, Patriquin CJ, Pham HP, Sanchez AP, Schneiderman J, Witt V, Zantek ND, Dunbar NM (2023) Guidelines on the use of therapeutic apheresis in clinical practice - evidence-based approach from the writing committee of the American Society for Apheresis: the ninth special issue. J Clin Apher 38:77–278 37017433 10.1002/jca.22043

[53] Larsen FS, Schmidt LE, Bernsmeier C, Rasmussen A, Isoniemi H, Patel VC, Triantafyllou E, Bernal W, Auzinger G, Shawcross D, Eefsen M, Bjerring PN, Clemmesen JO, Hockerstedt K, Frederiksen HJ, Hansen BA, Antoniades CG, Wendon J (2016) High-volume plasma exchange in patients with acute liver failure: an open randomised controlled trial. J Hepatol 64:69–78 26325537 10.1016/j.jhep.2015.08.018

[54] Zoica BS, Deep A (2021) Extracorporeal renal and liver support in pediatric acute liver failure. Pediatr Nephrol 36:1119–1128 32500250 10.1007/s00467-020-04613-4

[55] Deville K, Charlton N, Askenazi D (2023) Use of extracorporeal therapies to treat life-threatening intoxications. Pediatr Nephrol 39:105–113 36988694 10.1007/s00467-023-05937-7

[56] Levy G, Yaffe SJ (1974) Relationship between dose and apparent volume of distribution of salicyiate in children. Pediatr Res 8:365–365 4431669

[57] Meyer TW (2012) The removal of protein-bound solutes by dialysis. J Ren Nutr 22:203–206 22200443 10.1053/j.jrn.2011.10.011

[58] Dudley MN, Shyu W, Nightingale C, Quintiliani R (1986) Effect of saturable serum protein binding on the pharmacokinetics of unbound cefonicid in humans. Antimicrob Agents Chemother 30:565–569 3789691 10.1128/aac.30.4.565 PMC176481

[59] Mirrakhimov AE, Barbaryan A, Gray A, Ayach T (2016) The role of renal replacement therapy in the management of pharmacologic poisonings. Int J Nephrol 2016:304329 10.1155/2016/3047329 PMC5155094 28042482

[60] Allawati H, Dallas L, Nair S, Palmer J, Thaikandy S, Hutchison C (2020) A pharmacokinetic study comparing the clearance of vancomycin during haemodialysis using medium cut-off membrane (theranova) and high-flux membranes (revaclear). Toxins 12:317 32408589 10.3390/toxins12050317 PMC7290329

[61] Tyagi PK, Winchester JF, Feinfeld DA (2008) Extracorporeal removal of toxins. Kidney Int 74:1231–1233 18974758 10.1038/ki.2008.476

[62] Weidhase L, Haussig E, Haussig S, Kaiser T, de Fallois J, Petros S (2019) Middle molecule clearance with high cut-off dialyzer versus high-flux dialyzer using continuous veno-venous hemodialysis with regional citrate anticoagulation: a prospective randomized controlled trial. PLoS One 14:e0215823 31026303 10.1371/journal.pone.0215823 PMC6485708

[63] Kane SL, Constantiner M, Staubus AE, Meinecke CD, Sedor JR (2000) High-flux hemodialysis without hemoperfusion is effective in acute valproic acid overdose. Ann Pharmacother 34:1146–1151 11054983 10.1345/aph.19387

[64] Sikma M, Van den Broek M, Meulenbelt J (2012) Increased unbound drug fraction in acute carbamazepine intoxication: suitability and effectiveness of high-flux haemodialysis. Intensive Care Med 38:916–917 22327559 10.1007/s00134-012-2501-8 PMC3332384

[65] Vilay AM, Mueller BA, Haines H, Alten JA, Askenazi DJ (2010) Treatment of methotrexate intoxication with various modalities of continuous extracorporeal therapy and glucarpidase. Pharmacotherapy 30:111 20030480 10.1592/phco.30.1.111

[66] Kıhtır HS, Yıldırım HM, Yeşilbaş O, Duramaz BB, Şevketoğlu E (2016) Single-pass albumin dialysis in a child aged six months with phenobarbital poisoning. Turk Pediatr Ars 51:228 10.5152/TurkPediatriArs.2016.2335 PMC5242253 28123338

[67] Chung YK, Chang KY, Park HS, Kim MH, Lee KM, Lim TS, Kim HW (2014) Severe carbamazepine intoxication unresponsive to albumin-enhanced continuous venovenous hemodiafiltration with low dialysate flow. Hemodial Int 18:551–555 24422855 10.1111/hdi.12132

[68] Koivusalo AM, Yildirim Y, Vakkuri A, Lindgren L, Höckerstedt K, Isoniemi H (2003) Experience with albumin dialysis in five patients with severe overdoses of paracetamol. Acta Anaesthesiol Scand 47:1145–1150 12969110 10.1034/j.1399-6576.2003.00190.x

[69] McIntyre CW, Fluck RJ, Freeman JG, Lambie SH (2002) Use of albumin dialysis in the treatment of hepatic and renal dysfunction due to paracetamol intoxication. Nephrol Dial Transplant 17:316–317 11812892 10.1093/ndt/17.2.316

[70] Pichon N, François B, Clavel M, Vignon P, Chevreuil C, Michel Gaulier J (2006) Albumin dialysis: a new therapeutic alternative for severe diltiazem intoxication. Clin Toxicol (Phila) 44:195–196 16615682 10.1080/15563650500516041

[71] Dobisova A, Vavrinec P, Vavrincova-Yaghi D, Gebhardtova A, Henning RH, Yaghi A (2021) Case report: enhanced diazepam elimination with the molecular adsorbents recirculating system (MARS) in severe autointoxication: a survival case report. Front Med (Lausanne) 8:189 10.3389/fmed.2021.633250 PMC8006414 33791324

[72] Covic A, Goldsmith DJ, Gusbeth-Tatomir P, Volovat C, Dimitriu AG, Cristogel F, Bizo A (2003) Successful use of Molecular Absorbent Regenerating System (MARS) dialysis for the treatment of fulminant hepatic failure in children accidentally poisoned by toxic mushroom ingestion. Liver Int 23:21–27 12950957 10.1034/j.1478-3231.23.s.3.9.x

[73] Prokurat S, Grenda R, Lipowski D, Kaliciński P, Migdal M, Prokurat S, Grenda R, Lipowski D, Kaliciński P, Migdal M (2002) MARS procedure as a bridge to combined liver–kidney transplantation in severe chromium–copper acute intoxication: a paediatric case report. Liver 22:76–77 12220311 10.1034/j.1600-0676.2002.00016.x

[74] Sen S, Ratnaraj N, Davies NA, Mookerjee RP, Cooper CE, Patsalos PN, Williams R, Jalan R (2003) Treatment of phenytoin toxicity by the molecular adsorbents recirculating system (MARS). Epilepsia 44:265–267 12558586 10.1046/j.1528-1157.2003.31402.x

[75] Korsheed S, Selby NM, Fluck RJ (2007) Treatment of severe theophylline poisoning with the molecular adsorbent recirculating system (MARS). Nephrol Dial Transplant 22:969–970 17164319 10.1093/ndt/gfl640

[76] Soni KD, Singh A, Tyagi A, Singh Y, Aggarwal R, Trikha A (2023) Risk factors and outcomes of post-traumatic acute kidney injury requiring renal replacement therapy: a case-control study. Indian J Crit Care Med 27:22–25 36756485 10.5005/jp-journals-10071-24380 PMC9886052

[77] Harrois A, Libert N, Duranteau J (2017) Acute kidney injury in trauma patients. Curr Opin Crit Care 23:447–456 29035925 10.1097/MCC.0000000000000463

[78] Harrois A, Soyer B, Gauss T, Hamada S, Raux M, Duranteau J (2018) Prevalence and risk factors for acute kidney injury among trauma patients: a multicenter cohort study. Crit Care 22:344 30563549 10.1186/s13054-018-2265-9 PMC6299611

[79] Yousefifard M, Toloui A, Forouzannia SA, Ataei N, Hossein H, Zareie Shab Khaneh A, Karimi Ghahfarokhi M, Jones ME, Hosseini M (2022) Prevalence and mortality of post-traumatic acute kidney injury in children; a systematic review and meta-analysis. Arch Acad Emerg Med 10:e89 36590654 10.22037/aaem.v10i1.1660 PMC9795413

[80] Beitland S, Os I, Sunde K (2014) Primary injuries and secondary organ failures in trauma patients with acute kidney injury treated with continuous renal replacement therapy. Scientifica (Cairo) 2014:235215 25587490 10.1155/2014/235215 PMC4284970

[81] Beitland S, Moen H, Os I (2010) Acute kidney injury with renal replacement therapy in trauma patients. Acta Anaesthesiol Scand 54:833–840 20528778 10.1111/j.1399-6576.2010.02253.x

[82] Perkins ZB, Captur G, Bird R, Gleeson L, Singer B, O’Brien B (2019) Trauma induced acute kidney injury. PLoS One 14:e0211001 30682106 10.1371/journal.pone.0211001 PMC6347290

[83] Karvellas CJ, Farhat MR, Sajjad I, Mogensen SS, Leung AA, Wald R, Bagshaw SM (2011) A comparison of early versus late initiation of renal replacement therapy in critically ill patients with acute kidney injury: a systematic review and meta-analysis. Crit Care 15:R72 21352532 10.1186/cc10061 PMC3222005

[84] Gettings LG, Reynolds HN, Scalea T (1999) Outcome in post-traumatic acute renal failure when continuous renal replacement therapy is applied early vs. late. Intensive Care Med 25:805–813 10447537 10.1007/s001340050956

[85] Li X, Bai M, Yu Y, Ma F, Zhao L, Li Y, Wu H, Zhou L, Sun S (2022) Earlier continuous renal replacement therapy is associated with reduced mortality in rhabdomyolysis patients. Ren Fail 44:1743–1753 36259466 10.1080/0886022X.2022.2132170 PMC9586620

[86] Jerman A, Andonova M, Persic V, Gubensek J (2022) extracorporeal removal of myoglobin in patients with rhabdomyolysis and acute kidney injury: comparison of high and medium cut-off membrane and an adsorber cartridge. Blood Purif 51:907–911 35340002 10.1159/000521923 PMC9808672

