# Supplementary material for: Extracorporeal pediatric renal replacement therapy: diversifying application beyond kidney failure
Source: Pediatr Nephrol. 2024 Oct 8;40(4):923–32. doi: 10.1007/s00467-024-06533-z (PMC11885324; doi:10.1007/s00467-024-06533-z)
Supplement: Supplementary file 1 — Graphical abstract (PPTX 646 KB) [file 467_2024_6533_MOESM1_ESM.pptx]

## Slide 1
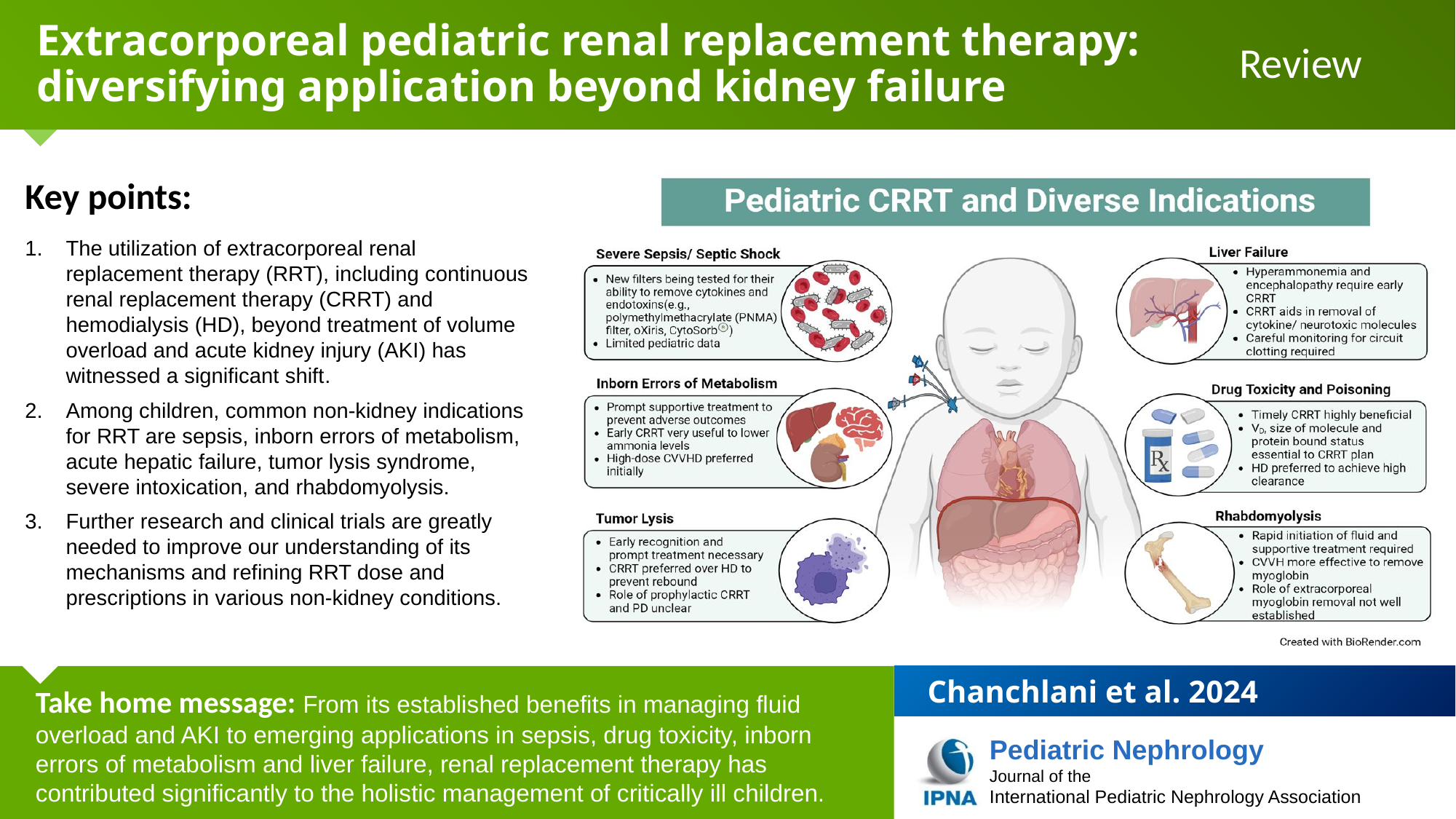

Extracorporeal pediatric renal replacement therapy:
diversifying application beyond kidney failure
Key points:
The utilization of extracorporeal renal replacement therapy (RRT), including continuous renal replacement therapy (CRRT) and hemodialysis (HD), beyond treatment of volume overload and acute kidney injury (AKI) has witnessed a significant shift.
Among children, common non-kidney indications for RRT are sepsis, inborn errors of metabolism, acute hepatic failure, tumor lysis syndrome, severe intoxication, and rhabdomyolysis.
Further research and clinical trials are greatly needed to improve our understanding of its mechanisms and refining RRT dose and prescriptions in various non-kidney conditions.
Consider including a representative figure or table from your review article, if relevant, and if you have the requisite permissions.
Chanchlani et al. 2024
Take home message: From its established benefits in managing fluid overload and AKI to emerging applications in sepsis, drug toxicity, inborn errors of metabolism and liver failure, renal replacement therapy has contributed significantly to the holistic management of critically ill children.
